# Radioiodine remnant ablation in low-risk differentiated thyroid cancer patients who had R0 dissection is an over treatment

**DOI:** 10.1002/cam4.443

**Published:** 2015-03-09

**Authors:** Chandrasekhar Bal, Sanjana Ballal, Ramya Soundararajan, Saurav Chopra, Aayushi Garg

**Affiliations:** 1 Department of Nuclear Medicine, All India Institute of Medical SciencesNew Delhi, 110029, India; 2 Medical Student, All India institute of Medical SciencesNew Delhi, 110029, India

**Keywords:** Differentiated thyroid cancer, dynamic risk-stratification, low-risk, radioiodine therapy, remnant ablation

## Abstract

Low-risk (LR) differentiated thyroid cancer (DTC) patients should be ablated or not, albeit, with small dose of radioiodine is highly controversial. We hypothesized that those LR DTC patients who were surgically ablated need no radioiodine remnant ablation (RRA). This study aims to evaluate the long-term outcome in these two groups of patients. Retrospective cohort study conducted from January 1991 to December 2012. Based on extent of surgical resection and histopathology, LR DTC patients were classified as Gr-1: 169 patients, who were surgically ablated; Gr-2: 153 patients, who had significant remnant in thyroid bed. Basal parameters were comparable between two groups except pretherapy 24 h radioiodine uptake (0.16 ± 0.01% vs. 5.64 ± 0.46%; *P* < 0.001). No patient received RRA in Gr-1; Gr-2 patients were administered 30 mCi ^131^I. Total number of events (recurrence, persistent, and progression of disease), with median follow up of 10.3 years, was observed in 10/322 (3.1%) of LR DTC patients. Only one patient had disease recurrence from Gr-1, who became disease-free after radioiodine therapy. Similarly, one patient from 126, who was ablated with single dose of RRA, had recurrence from Gr-2. However, 8/27 (29.7%) patients from Gr-2 had persistent disease; even two of them subsequently developed disease progression, who failed first-dose of RRA. The event-free survival rates were 99.4% and 94.1% (*P* = 0.006) in Gr-1 and Gr-2, respectively. *RRA* is an overtreatment in surgically ablated LR DTC patients. Successfully ablated RRA patients also had similar long-term outcome, however, those who failed, should be re-stratified as intermediate-risk category, and managed aggressively.

## Introduction

Thyroid remnant tissue is any normal thyroid tissue or microscopic disease in the thyroid bed left out after total/near-total thyroidectomy (NTT). Radioiodine remnant ablation (RRA) is defined as ablation of this remnant thyroid tissue by administration of radioiodine (RAI) 1. RRA has been accepted as the standard-of-care in the management of differentiated thyroid cancer (DTC) 2. Nevertheless, achievement of successful surgical ablation is considered as the eutopic goal where no further RRA is required 3. While in most of the patients with significant remnant thyroid tissue demonstrated on diagnostic ^131^I whole body scan (Dx-WBS)/Ultrasonography (USG) of neck/raised stimulated-thyroglobulin (sTg > 10 ng/mL), RRA is administered with the aim to achieve complete tissue ablation. Proponents of RRA argue that remnant ablation, (a) reduces the chances of recurrence by targeting the microscopic tumor foci, (b) provides an added advantage by increasing the sensitivity of Dx-WBS, and (c) simplifies the follow up by serial estimation of serum thyroglobulin (Tg) levels to detect recurrence 2.

According to the American Thyroid Association (ATA) Revised Guidelines, DTC with low-risk (LR) of recurrence is defined as disease limited only to thyroid without extrathyroidal extension, stage I and II (T1N0M0, T2N0M0) if patient age >45 years and stage I (Any T,N0M0) if patient age <45 years [T1—primary tumor diameter 2 cm or smaller, T2—primary tumor diameter >2–4 cm), no local (N0) and distant metastases (M0)], having no aggressive histologic subtype, no macroscopic remnant after surgery, and no radioiodine concentration outside the thyroid bed (N0) in postoperative Dx-WBS 2. ATA guideline also recommends that the patients having (a) unifocal classical papillary carcinoma, (b) tumor size less than 2 cm, and (c) without adverse histologic variant may be exempted from RRA 2. In other words, vast majority of DTC patients need RRA. Recently, several authors have raised questions on the indiscriminate use of RRA in LR DTC patients 3,4. There are growing evidences suggesting dose-related harmful side-effects of RAI therapy; a risk-based management approach has been advocated for the treatment of LR DTC 5.

Total thyroidectomy (TT) or surgical ablation although is desired, but practical achievement is rather low, in the hands of low volume thyroid surgeons. Surgical ablation rate in our institute was 7% till the year 2000, increased to 10% till 2009 and currently this rate has further increased to 15% after the revised ATA guidelines was published in 2009 recommending TT in all DTC patients with tumor size greater than 1 cm. If the surgeons could not achieve TT and leave significant remnant tissue with radioiodine uptake (RAIU) greater than 0.2% and/or sTg > 10 ng/mL, we regularly advocate for remnant ablation with 1110 MBq of ^131^I. Thus, we have two comparable groups of LR patients, one with ≤0.2% RAIU at 24 h and no RRA done; the other with greater than 0.2% uptake and undergone RRA with 1110 MBq ^131^I.

An important study called “IoN trial” is currently undertaken by 32 centers from UK, where surgically ablated (R0) LR DTC patients are recruited and randomized to either receive low-dose (1110 MBq) RRA or no RRA 6. We feel this trial, shall probably able to throw light on surgically ablated (R0) patient, whether need RRA or not, but not be able to answer, whether RRA is necessary or not in patients with significant remnant tissue present. In our practice, we consider administering radioiodine in surgically ablated LR DTC patients is an over treatment. As an institutional policy we do not administer radioiodine in surgically ablated patients but only put them on levothyroxine dose to keep their thyroid-stimulating hormone (TSH) level between 0.5 and 1.0 *μ*IU/mL. However, the question remains unanswered “whether the LR DTC patients (a) surgically ablated, or (b) with significant remnant present, need RRA or not”. In this light, we tried to analyze our cohort to find the significance of RRA and its long-term outcome in LR DTC patients.

## Materials and Methods

### Patients and study

All India Institute of Medical Sciences, New Delhi is a tertiary care federally funded teaching institution, delivering health care to whole of northern India. Thyroid clinic has a database of about 6500 thyroid cancer patients and regularly following all these patients since 1967 onwards. Currently, we are treating approximately 700 newly diagnosed thyroid cancer patients annually. This study is a retrospective analysis, involving DTC patients treated between January 1991 and December 2012 at our center. The study was approved by the institute ethics committee.

### Patient inclusion and exclusion criteria

As per ATA guideline the DTC patients with LR of recurrence that is the patients with stage I and II if patient age >45 years and stage I if patient age <45 years, all macroscopic tumor resected, no locoregional invasion of tumor and/aggressive histology were included for the analysis 2. From the medical records of all the patients treated during this period, the data of 326 patients with LR DTC were extracted.

**Figure 1:**
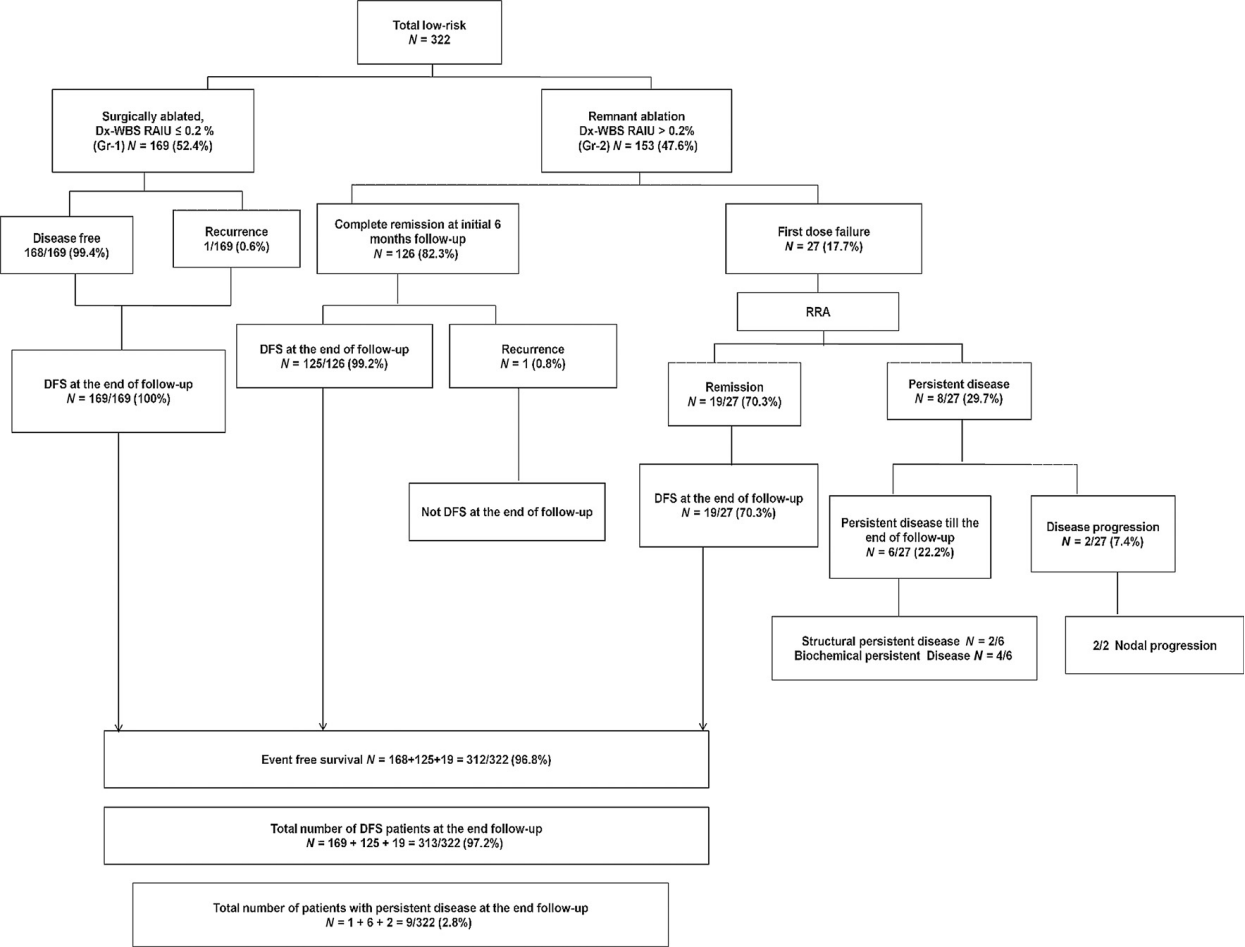
Clinical course of patients in the surgically ablated group (Gr-1) and radioiodine remnant ablation group (Gr-2). Dx-WBS, diagnostic ^131^I whole body scan; DFS, disease-free survival; RAIU, radioiodine uptake; RRA, radioiodine remnant ablation.

Patients who underwent less than NTT, aggressive histology and follow up <12 months were excluded from the study. Four patients with inadequate follow up were excluded. Finally, 322 patients with LR DTC were included in the study. Figure1 depicts the clinical profile of this study.

### Diagnosis and treatment protocol

The standard protocol of treatment in DTC patients followed at our institution was NTT or TT. As a policy, all DTC patients undergo Dx-WBS for proper scintigraphic staging, before any RAI therapy is planned. The completeness of surgical resection is assessed by the Dx-WBS (74–111 MBq), performed 25–35 days after surgery without thyroxine supplementation. Patients showing no uptake in thyroid bed or extrathyroidal site in Dx-WBS, RAIU ≤ 0.2%, sTg ≤ 10 ng/mL, and anti-Tg antibody negative were considered as surgically ablated (Gr-1) and were not administered RRA therapy. If Dx-WBS reveals significant remnant, extrathyroidal uptake, nodal/distant metastatic deposit then we ablate/treat with RAI. Patients with significant thyroid bed uptake, RAIU > 0.2% and stimulated Tg > 10 ng/mL constitute Gr-2 and were treated with 1110 MBq of ^131^I.

After administration of RRA, all patients were admitted in an isolated RAI therapy ward for 24 h and were discharged when the radiation levels dropped below the permissible environmental level of radiation (50 *μ*Sv/h) as per National Regulatory guidelines. Appropriate radiation safety counseling was given to all patients. The posttherapeutic scans were performed at the time of discharge to look for the additional sites of disease. Thereafter, all patients were prescribed once daily 2 *μ*g/kg of levothyroxine in empty stomach before breakfast in order to maintain TSH between 0.5 and 1.0 *μ*IU/mL.

### Follow up

Among the patients who received RRA, initial follow up was performed at 6–9 months later after 4 weeks of levothyroxine withdrawal. The patients undergo serum Tg, anti-Tg antibody (ATA) and TSH assays along with Dx-WBS and 24 h RAIU. The patients who achieved complete ablation as per our institutional protocol were on regular follow ups annually for the detection of disease relapse. In patients, who failed to achieve ablation at the initial follow-up study multiple doses of RAI were administered ranging from 1110 to 3700 MBq until achieved complete remission. Once complete ablation was achieved, long-term annual check-ups were followed in the form of routine physical examination, USG examination of neck, thyroid profile, Tg and ATA assays. In cases of suspected recurrence, structural and functional imaging investigations like chest-computed tomography (CT) scans, ^131^I Dx-WBS, ^18^F-Fluorodeoxyglucose (FDG) positron emission tomography (PET)/CT scans were ordered, wherever it was deemed necessary.

### Definition of outcome endpoints

Disease status was stratified as disease-free, biochemical or structural persistent disease, recurrence and progression based on the scintigraphic findings on Dx-WBS, serum Tg levels, and structural imaging (USG, CT and PET/CT). *Remission* was defined as sTg levels ≤10 ng/mL 7,8 along with negative Dx-WBS and 24 h RAIU ≤0.2%. A patient was considered as *disease-free* if he/she stayed in remission till the last follow up. Patients with constantly elevated, although static, serum Tg levels >10 ng/mL in the absence of any structural lesions were taken as *biochemical persistent disease*. Patients with constantly elevated serum Tg with accompanying structural lesions were considered as *structural persistent disease*. *Recurrence* was defined as reappearance of disease at least 12 months after initial documented remission. Appearance of new lesion(s) in Dx-WBS or on structural imaging in a patient who had never achieved remission was defined as *progression*. An event was considered as persistent disease or reappearance of documented locoregional/distant disease or disease progression with increase in the serum Tg levels and/or demonstration of structural/functional lesions.

### Statistical analysis

Univariate analysis was used to compare the disease characteristics of both patients groups (Gr-1 vs. Gr-2). Univariate analysis was also done among the patients who achieved remission and who did not achieve remission at the initial follow up and at the end of follow up. Continuous variables were calculated as mean, median, standard deviation (SD), and standard error of mean (SEM). Categorical variables were compared by chi-square test or the Fisher’s exact test on the basis of expected cell frequencies. Among the latter group of patients, factors associated with failure to achieve remission were analyzed. *P*-values <0.05 were considered as significant. Stata 11.2 (StataCorp, College Station, TX) was used for the analysis.

## Results

The median follow-up duration was 10.3 years (range, 1–20 years). None of the patients had family history of DTC or history of prior radiation exposure. All patients underwent TT or NTT. Nodal dissection was performed in only 43 (13.4%) patients and all were negative for nodal metastasis. All these were central compartment neck dissection. Among the total 322 patients with LR DTC, 169 (52.4%) (mean age 35.43 ± 6.59 years, females—79.3%, papillary histology—92.4%, mean 24 h RAIU 0.16 ± 0.01%), belonging to Gr-1 received no RRA, whereas the remaining 153 (47.7%) (mean age 36.78 ± 7.2 years, females—75.8%, papillary 86.9%, mean 24 h RAIU 5.64 ± 0.46%) patients (Gr-2) were administered 1110 MBq ^131^I. The comparison of baseline clinical characteristics of patients between Gr-1 and Gr-2 are depicted in Table1. The two groups were found to be similar with respect to all the baseline characteristics except for pretherapy RAIU values (*P* < 0.001) for obvious reason.

**Table 1:** Characteristics of patients who became surgically ablated (Gr-1) and patients who received RRA (Gr-2)

Variables	Surgically ablated (Gr-1), *N* = 169	RRA treatment group (Gr-2), *N* = 153	*P*-value
Sex			
Male	35 (20.7)	37 (24.2)	0.47
Female	134 (79.3)	116 (75.8)	
Preoperative metabolic status			
Euthyroid	164 (97)	150 (98.0)	0.611
Hypothyroid	4 (2.4)	3 (2.0)	
Hyperthyroid	1 (0.6)	0 (0.0)	
Nodal dissection			
No	144 (85.2)	135 (88.2)	0.413
Yes	25 (14.8)	18 (11.8)	
Histology			
Papillary	156 (92.4)	133 (86.9)	0.116
Follicular	13 (7.6)	20 (13.1)	
Completion thyroidectomy			
No	118 (69.7)	105 (68.6)	0.844
Yes	51 (30.3)	48 (31.4)	
T			
1	68 (40)	70 (45.8)	0.288
2	101 (60)	83 (54.2)	
TNM stage			
Stage I	151 (89.4)	128 (83.7)	0.139
Stage II	18 (10.6)	25 (16.3)	
24 h RAIU % (Mean ± SEM)	0.16 ± 0.01 (0.15–0.18)	5.64 ± 0.46 (0.3–16.0)	0.001

Data are expressed as number (percentage), unless otherwise specified. *P* < 0.05 was considered significant. RRA, radioiodine remnant ablation; RAIU, radioiodine uptake; SEM, standard error of mean.

### Results of RRA

Among the Gr-2 who received RRA, 126 (82.3%) patients achieved successful ablation at the initial 6–9 months follow up, whereas 27 (17.7%) patients failed to achieve ablation of remnant. The factors associated with failure to achieve remission with single dose of RRA were analyzed. None of the disease related factors were found to be significantly associated with reduced odds of remission except for the 24 h RAIU value of 4.59 ± 0.45% versus 7.81 ± 1.54%, *P* < 0.01 (Table2). On subsequent therapies, 19/27 (70.3%) patients achieved ablation in due course of time, however, eight patients continued to be living with persistent disease; subsequently, two even progressed as non RAI concentrating nodal disease.

**Table 2:** Characteristics of patients who received RRA therapy stratified according to response to RRA therapy at initial 6 months follow up

Variable	Initial follow-up Dx-WBS remission achieved (*N* = 126)	Initial follow-up Dx-WBS persistent disease observed (*N* = 27)	*P*-value
Age			
<45 years	95 (75.4)	20 (74.1)	0.885
≥45 years	31 (24.6)	7 (25.9)	
Sex			
Male	30 (23.8)	7 (25.9)	0.816
Female	96 (76.2)	20 (74.1)	
Preoperative metabolic status			
Euthyroid	124 (98.4)	26 (96.3)	0.472
Hypothyroid	2 (1.6)	1 (3.7)	
Nodal dissection			
No	110 (87.3)	25 (92.6)	0.742
Yes	16 (12.7)	2 (7.4)	
Histology			
Papillary	112 (88.9)	21 (77.8)	0.120
Follicular	14 (11.1)	6 (22.2)	
Completion thyroidectomy			
No	83 (65.9)	22 (81.5)	0.113
Yes	43 (34.1)	5 (18.5)	
T stage			
T1	57 (45.2)	13 (48.2)	0.783
T2	69 (54.8)	14 (51.8)	
TNM stage			
Stage I	106 (84.1)	22 (81.5)	0.736
Stage II	20 (15.9)	5 (18.5)	
24 h RAIU % (Mean ± SEM)	4.59 ± 0.45% (3.68–5.5)	7.8 ± 1.54% (4.62–11.05)	0.01

Data are expressed as number (percentage), unless otherwise specified*. P* < 0.05 was considered significant. RRA, radioiodine remnant ablation; Dx-WBS, Diagnostic ^131^I whole body scan; RAIU, radioiodine uptake; SEM, standard error of mean.

### Final outcome

With median follow up of 10.3 years, 168 (99.4%) patients from Gr-1 had never developed any event. Only a 21-year-old female patient in Gr-1 (*n* = 169) developed nodal recurrence (0.6%) at 14 months. She was administered 1850 MBq (50 mCi) of ^131^I and was ablated with single dose of RAI. She was disease-free by the end of follow up. While, in Gr-2 among 126 patients who were successfully ablated with single dose of RRA, one developed recurrence. From Gr-2, 144/153 (94.1%) achieved complete remission, and 9 (5.9%) patients did not achieve remission in spite of multiple doses of RAI [1 recurrence, six persistent diseases (four biochemical, two structural), and two progressions in the form of nodal disease]. Univariate analysis between the remission (*n* = 144) and nonremission (*n* = 9) in Gr-2 at the end of follow up was analyzed and depicted in Table3. None of variables could predict recurrence/persistent disease.

**Table 3:** Characteristics of patients who RRA therapy stratified according to response to RRA therapy at end follow up

Variable	Remission at end of follow up (N = 144)	No remission till end of follow up (N = 9)	*P*-value
Age			
<45 years	114 (79.2)	5 (55.6)	0.111
≥45 years	30 (20.8)	4 (44.4)	
Sex			
Male	35 (24.3)	2 (22.2)	0.887
Female	109 (75.7)	7 (77.8)	
Preoperative metabolic status			
Euthyroid	141 (97.9)	9 (100.0)	1.000
Hypothyroid	3 (2.1)	0 (0)	
Nodal dissection			
No	127 (88.2)	8 (88.9)	0.950
Yes	17 (11.8)	1 (11.1)	
Histology			
Papillary	125 (86.8)	8 (88.9)	0.857
Follicular	19 (13.2)	1 (11.1)	
Completion thyroidectomy			
No	97 (67.4)	8 (88.9)	0.177
Yes	47 (32.6)	1 (11.1)	
T Stage			
T1	66 (45.8)	4 (44.4)	0.935
T2	78 (54.2)	5 (55.6)	
TNM stage			
Stage I	122 (84.7)	6 (66.7)	0.155
Stage II	22 (15.3)	3 (33.3)	
24 h RAIU % (Mean ± SEM)	5.1 ± 0.48 (4.15–6.07)	4.7 ± 1.19 (1.93–7.60)	0.8664

Data are expressed as number (percentage), unless otherwise specified. *P* < 0.05 was considered significant. RRA, radioiodine remnant ablation; RAIU, radioiodine uptake; SEM, standard error of mean.

### Survival analysis

The overall survival of patients was 100% as no patient died of thyroid cancer, and disease-free survival for LR patients in our series was 97.2% (313/322). The overall event-free survival was 96.8% (312/322). The event-free survival was 99.4% in the surgically ablated patients and 94.1% in RRA patients (*P* = 0.006).

## Discussion

There is unfortunately no randomized control trial (RCT) available in the literature, on long-term outcome of LR DTC, which could guide the thyroidologist to take evidence-based decision on RRA. This is partly because of low event rates—low recurrence and almost negligible mortality among the LR DTC patients—the role of RAI therapy in the treatment of DTC remains controversial. The current guideline is based, mostly on retrospective data, published from various high-volume thyroid cancer treating centers/institutions. Before the concept of risk-stratification was advocated in DTC, a high dose of RAI therapy was administered to all thyroid cancer patients following thyroidectomy, which was associated with increased risk of side-effects and probability of second malignancies 9. Due to the unclear risk–benefit ratio of high-dose RAI therapy in LR DTC patients several prospective randomized control studies evaluated the effectiveness of low-dose RAI therapy. In 1996, a RCT was published on 149 LR DTC patients from our institution, showed no significant difference in the success rate of remnant ablation between 1110 and 3700 MBq. We advocated 925–1850 MBq ^131^I remnant ablation in all LR DTC patients 10. Subsequent two more studies from our institution in 509 patients and in 422 patients reconfirmed the initial observation 11,12. Till date 15 RCTs have been published on this issue. Three meta-analyses have compiled all randomized trials, the effectiveness of 1110 versus 3700 MBq in LR patients, and all of them have concluded that low dose of radioiodine is as effective as high-dose RRA in LR DTC patients 13–15. Though the immediate outcome of successful ablation with 1110 versus 3700 MBq was published, however, long-term outcomes were not available with 1110 MBq RRA.

We regularly give 1110 MBq of ^131^I to our LR DTC patients, since 1990, for RRA. If we have to compare the long-term outcome, that is, recurrence and death in this group of patients given 1110 MBq of ^131^I, then comparison against a LR group not given RAI should be done. Hence, this follow-up study was to answer about the necessity of RRA and its long-term outcome in LR DTC patients. Conflicting data from some retrospective long-term outcome study, reported lower recurrence rates and improved overall survival with the use of RRA in LR DTC patients 16, many others have failed to observe any benefit with RRA 3,4. Some even seriously question the concept of RRA in LR DTC patients, and argue against RRA for its harmful dose-related short-term and long-term consequences of radioiodine in these patients 17–22. The part of the confusion was due to mixed bag of LR DTC patients as all of them were given RRA without doing pretherapy Dx-WBS to assess the size/mass of the remnant. Similar to van Nostrand et al. our institutional protocol is to do a routine pretherapy Dx-WBS for all patients referred for RRA 23. The vast majority of patients with intrathyroidal tumor (T1&T2), operated by high-volume thyroid surgeons, shall have no remnant tissue or negligible remnant; we strongly believe that those patients do not need any RRA. Whereas patients with significant thyroid remnant, whether need RRA or not, have to be separated first from LR patients with no remnant tissue demonstrated by WBS/RAIU/sTg and then assessed for long-term outcome. This study is a unique endeavor, with clear objectives, to answer the need of radioiodine as an adjuvant therapy and the long-term outcome in DTC patients who are at LR of recurrence and compared against similar number of patients who were surgically ablated and not given RAI.

The total event rate, that is, recurrence, persistent disease, and progression together in Gr-2 patients was 5.9% (9/153), and in Gr-1 only one patient 0.6% (1/169) developed nodal recurrence (*P* = 0.006). The current results validated our hypothesis that RRA in surgically ablated patient is an over treatment. The overall event rate, with median follow up of 10.3 years in our series of 322 patients, was found to be extremely low 3.2% (10/322). We strongly believe, the “IoN trial” whose result is expected in the year 2021, shall concur with our result 6. Similarly, observational retrospective studies published by Hay et al. 24 and Hundahl et al. 25 concluded that RRA does not decrease the recurrence rate after surgical ablation. However, Mazaferri et al. 16 had made different conclusion. They reported two groups: Group1, 230 patients who had undergone RRA and Group2, 789 treated with levothyroxine alone. With a median follow up of 18.9 years, the recurrence rate with levothyroxine alone was twofold 101/789 (12.8%; *P* < 0.0001) and the rate of distant relapse was threefold 19/789 (2.4%; *P* < 0.02), compared to that of patients given RRA in whom the recurrence rate was 15/230 (6.5%) and distant relapse was 2/230 (0.87%). We strongly presume that the patients treated and followed up by these authors at OSU, unlike Mayo or MSKCC, were mixed bag, thus giving varied result not reproduced by any other North American centers.

Achievement of remission should be an important goal in treating DTC patients with LR of recurrence. Considering the second question that patient with significant remnant whether need RRA or not: among Gr-2 (*n* = 153) patients undergoing RRA, 126 (82.3%) achieved complete ablation after first-dose of 1110 MBq ^131^I and 27 (17.7%) patients failed to achieve ablation at the initial follow up. None of the clinical baseline factors predicted the nonremission rate except for the mean pretherapy RAIU (*P* = 0.01), that is, higher the baseline RAIU, higher the failure rate. We observed that irrespective of the age, staging, and pathology of the tumor, patients with large volume of remnant were prone to have nonremission at initial follow up. One may argue, as we have administered only 1110 MBq for RRA in high RAIU group, higher dose probably could have better ablation rate. Elisei et al. 26 in 2009, even used fixed dose of 3700 MBq ^131^I for RRA, achieved remission rate of 82.3%, similar to our study at first follow up. Therefore, this study highlights the significance of achieving early remission and reconfirms our results with the previous finding of higher odds of persistent disease who failed to achieve remission with first-dose of RAI 27.

From this study, we have made an important observation, LR DTC patient with efficient surgery (Surgical Ablation), and the LR DTC patient who achieved first-dose successful RRA, had similar risk of recurrence (1/169 vs. 1/126; *P* = 0.61). Another important observation noted by us was that in Gr-2 patients given RRA therapy, 27 patients failed to achieve remission at initial follow up, however, eight patients failed to achieve remission, two even progressed, in spite of several doses of ^131^I administration. Further analyzing the event rate in Gr-2 patients, 1/126 (0.8%) recurred in successfully ablated patients versus 8/27 (29.7%) who failed first-dose ablation (*P* = 0.001). This highlights the fact that those patients who failed to achieve remission after RRA need to be dynamically risk-stratified to intermediate-risk category and be treated aggressively. From this study, we can conclude that patients with significant remnant need RRA.

However, as our study was a retrospective case-controlled in which bias cannot be eliminated, we need to undertake an RCT in LR DTC patients with significant remnant present (>0.2% to 5%) and randomized them into two groups, and one group should undergo RRA and other group no RRA, and follow them up for at least 10 years. If RRA has beneficial effect then event rate in “no RRA group” shall have significantly higher event rate than “RRA group”. The results of such a trial probably would give the definite answer regarding the true effect of RRA on the outcome in LR DTC patients with significant remnant in situ.

## Conclusion

We observed that surgically ablated patients with LR DTC do not need any RRA, for them it is an over treatment. However, if significant remnant tissue present in thyroid bed, successful remnant ablation had similar long-term outcome, like surgically ablated patients. The patients, who failed to achieve ablation with RRA, should be considered no more LR, and dynamically be risk-stratified as intermediate-risk, and managed aggressively.
